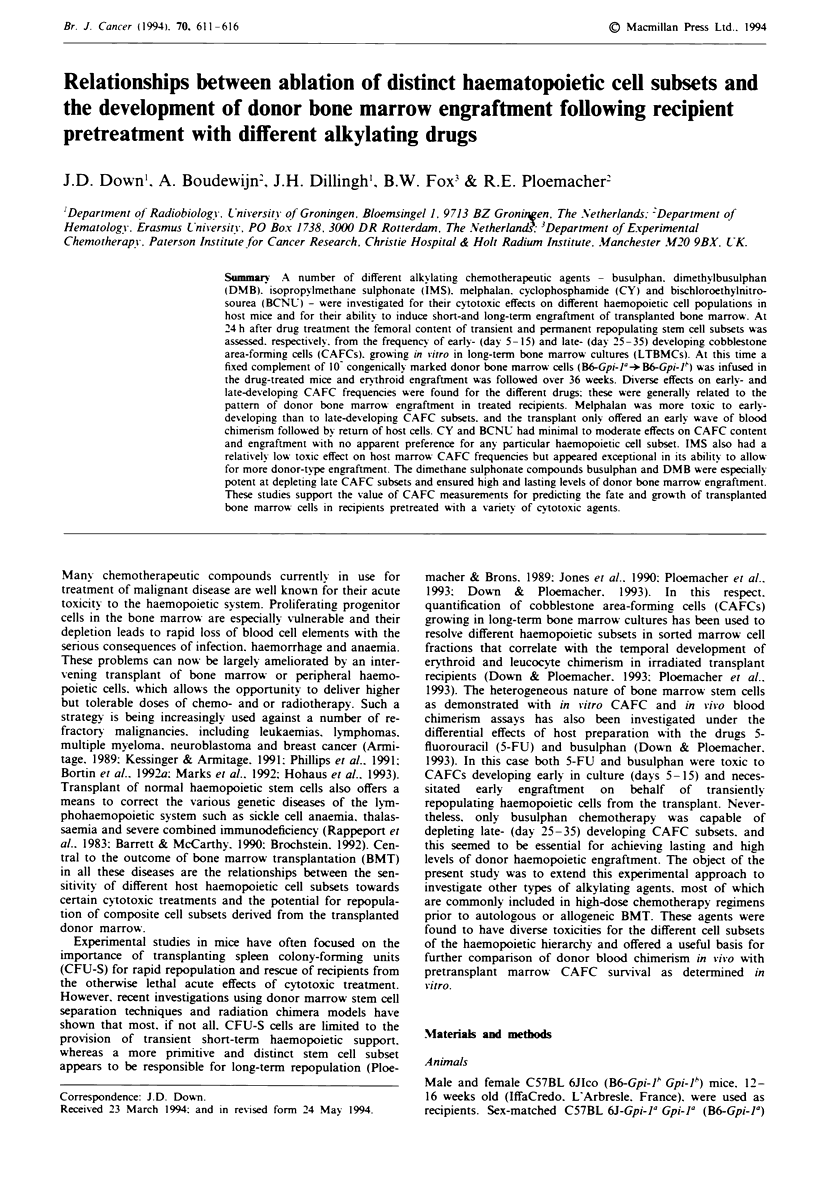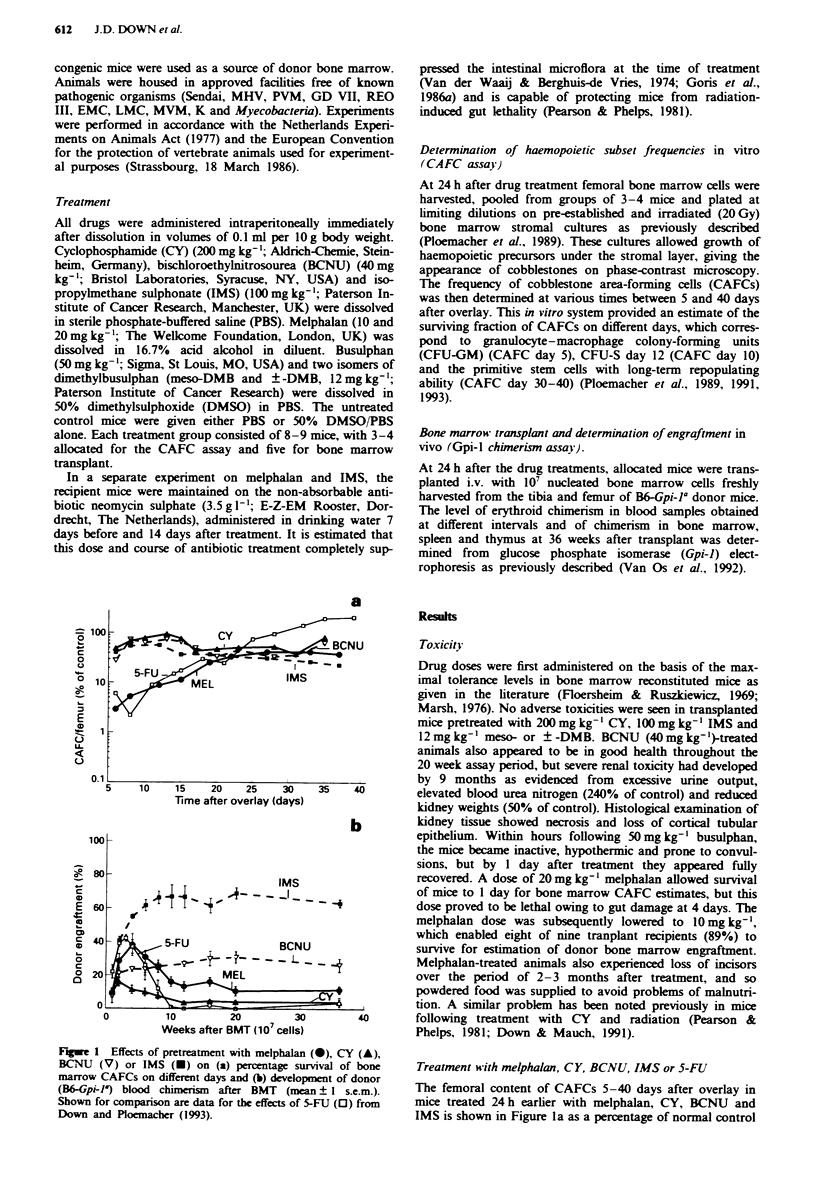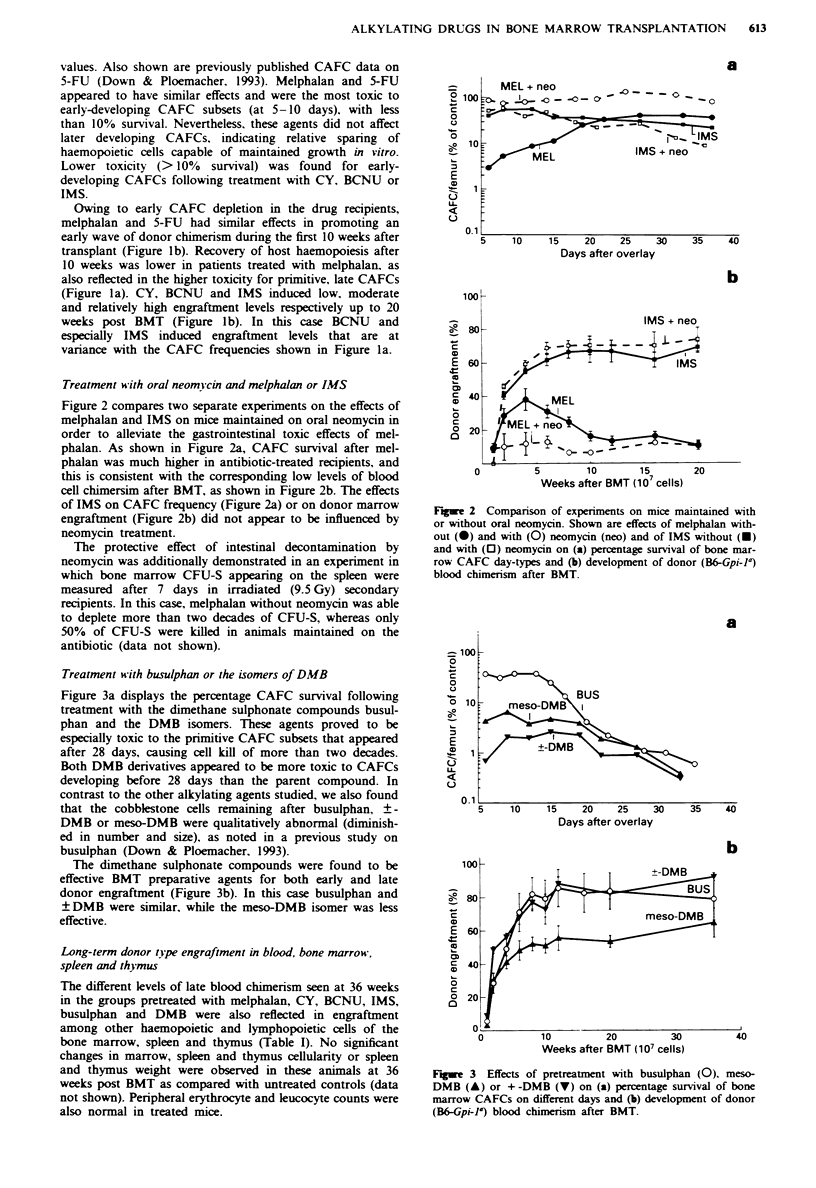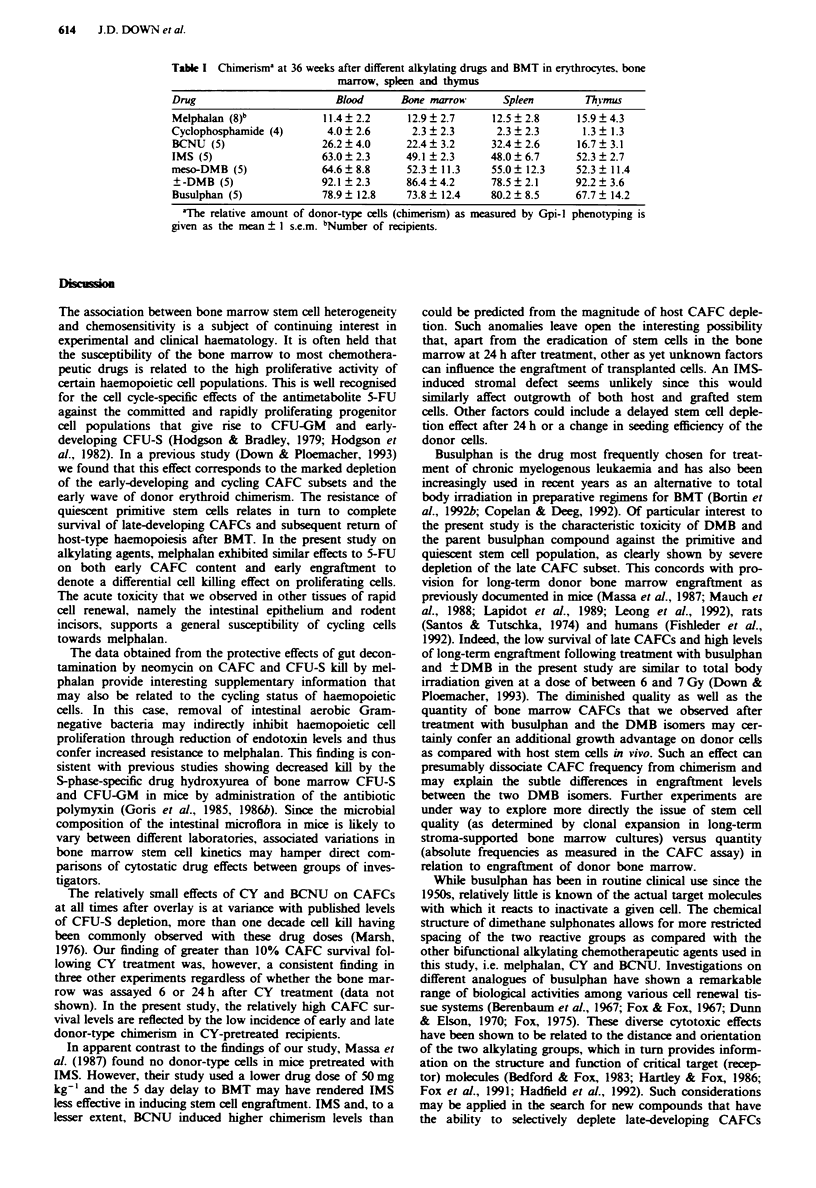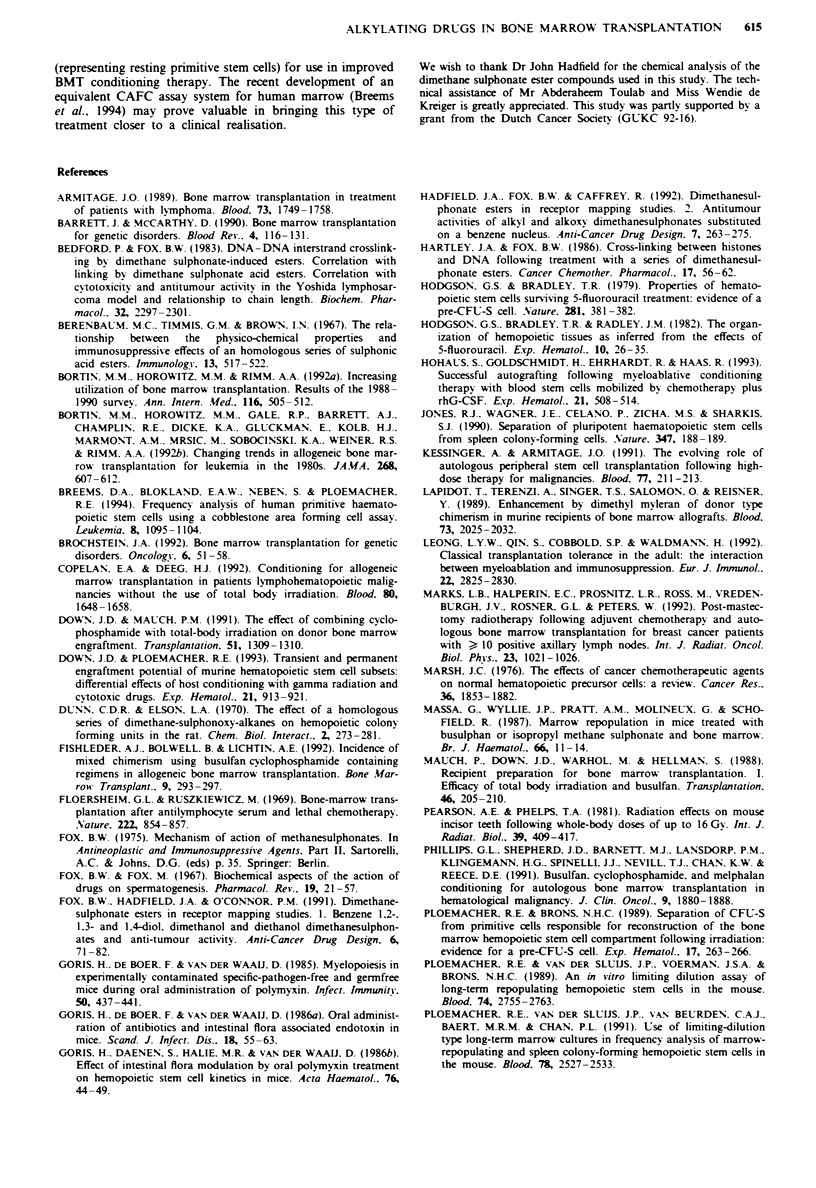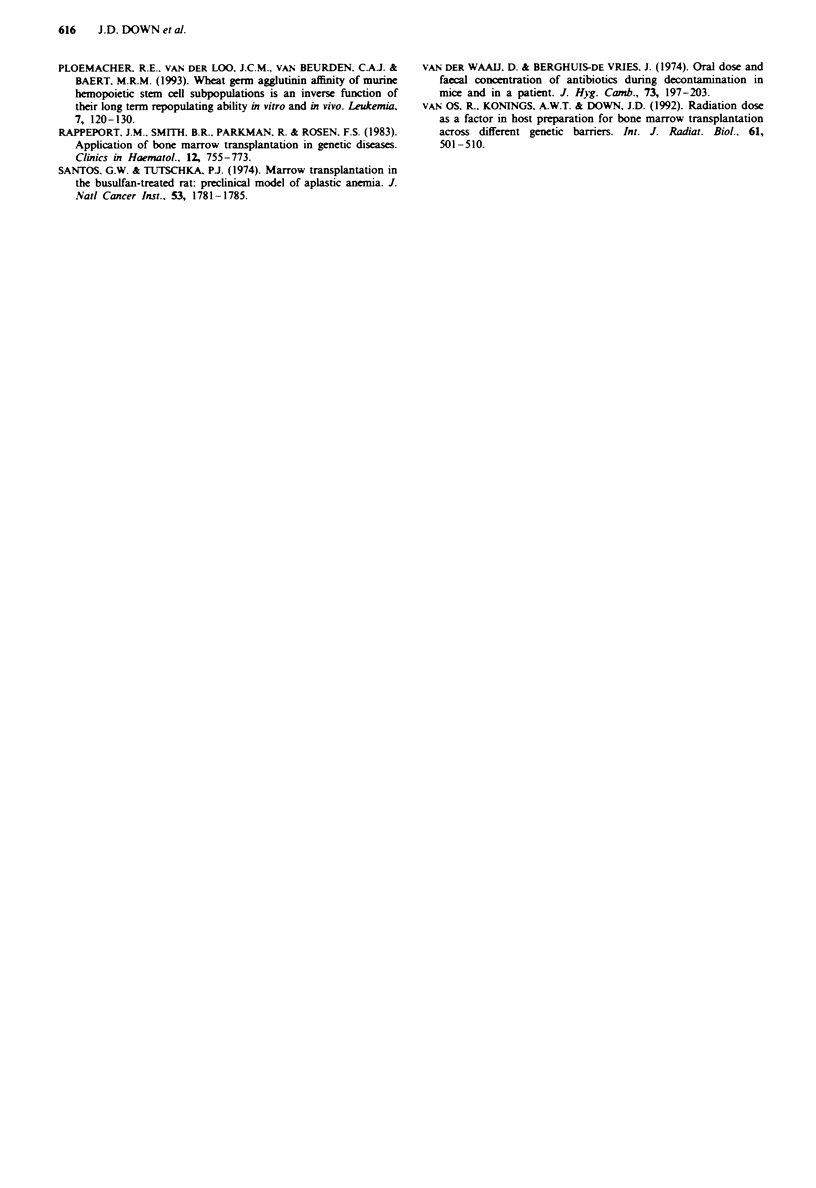# Relationships between ablation of distinct haematopoietic cell subsets and the development of donor bone marrow engraftment following recipient pretreatment with different alkylating drugs.

**DOI:** 10.1038/bjc.1994.359

**Published:** 1994-10

**Authors:** J. D. Down, A. Boudewijn, J. H. Dillingh, B. W. Fox, R. E. Ploemacher

**Affiliations:** Department of Radiobiology, University of Groningen, The Netherlands.

## Abstract

A number of different alkylating chemotherapeutic agents--busulphan, dimethylbusulphan (DMB), isopropylmethane sulphonate (IMS), melphalan, cyclophosphamide (CY) and bischloroethylnitrosourea (BCNU)--were investigated for their cytotoxic effects on different haemopoietic cell populations in host mice and for their ability to induce short- and long-term engraftment of transplanted bone marrow. At 24 h after drug treatment the femoral content of transient and permanent repopulating stem cell subsets was assessed, respectively, from the frequency of early- (day 5-15) and late- (day 25-35) developing cobblestone area-forming cells (CAFCs), growing in vitro in long-term bone marrow cultures (LTBMCs). At this time a fixed complement of 10(7) congenically marked donor bone marrow cells (B6-Gpi-1a-->B6-Gpi-1b) was infused in the drug-treated mice and erythroid engraftment was followed over 36 weeks. Diverse effects on early- and late-developing CAFC frequencies were found for the different drugs; these were generally related to the pattern of donor bone marrow engraftment in treated recipients. Melphalan was more toxic to early-developing than to late-developing CAFC subsets, and the transplant only offered an early wave of blood chimerism followed by return of host cells. CY and BCNU had minimal to moderate effects on CAFC content and engraftment with no apparent preference for any particular haemopoietic cell subset. IMS also had a relatively low toxic effect on host marrow CAFC frequencies but appeared exceptional in its ability to allow for more donor-type engraftment. The dimethane sulphonate compounds busulphan and DMB were especially potent at depleting late CAFC subsets and ensured high and lasting levels of donor bone marrow engraftment. These studies support the value of CAFC measurements for predicting the fate and growth of transplanted bone marrow cells in recipients pretreated with a variety of cytotoxic agents.


Br. J. Cancer (1994), 70, 611-616 © Macmillan Press Ltd., 1994

### Relationships between ablation of distinct haematopoietic cell subsets and the development of donor bone marrow engraftment following recipient pretreatment with different alkylating drugs

J.D. Down[1], A. Boudewijn[2], J.H. Dillingh[1], B.W. Fox[3] & R.E. Ploemacher[2]

[1]*Department of Radiobiology, University of Groningen, Bloemsingel 1, 9713 BZ Groningen, The Netherlands;* [2]*Department of Hematology, Erasmus University, PO Box 1738, 3000 DR Rotterdam, The Netherlands;* [3]*Department of Experimental Chemotherapy, Paterson Institute for Cancer Research, Christie Hospital & Holt Radium Institute, Manchester M20 9BX, UK.*

**Summary** A number of different alkylating chemotherapeutic agents – busulphan, dimethylbusulphan (DMB), isopropylmethane sulphonate (IMS), melphalan, cyclophosphamide (CY) and bischloroethylnitrosourea (BCNU) – were investigated for their cytotoxic effects on different haemopoietic cell populations in host mice and for their ability to induce short-and long-term engraftment of transplanted bone marrow. At 24 h after drug treatment the femoral content of transient and permanent repopulating stem cell subsets was assessed, respectively, from the frequency of early- (day 5–15) and late- (day 25–35) developing cobblestone area-forming cells (CAFCs), growing *in vitro* in long-term bone marrow cultures (LTBMCs). At this time a fixed complement of $10^7$ congenically marked donor bone marrow cells (B6-*Gpi-1*$^a$→B6-*Gpi-1*$^b$) was infused in the drug-treated mice and erythroid engraftment was followed over 36 weeks. Diverse effects on early- and late-developing CAFC frequencies were found for the different drugs; these were generally related to the pattern of donor bone marrow engraftment in treated recipients. Melphalan was more toxic to early-developing than to late-developing CAFC subsets, and the transplant only offered an early wave of blood chimerism followed by return of host cells. CY and BCNU had minimal to moderate effects on CAFC content and engraftment with no apparent preference for any particular haemopoietic cell subset. IMS also had a relatively low toxic effect on host marrow CAFC frequencies but appeared exceptional in its ability to allow for more donor-type engraftment. The dimethane sulphonate compounds busulphan and DMB were especially potent at depleting late CAFC subsets and ensured high and lasting levels of donor bone marrow engraftment. These studies support the value of CAFC measurements for predicting the fate and growth of transplanted bone marrow cells in recipients pretreated with a variety of cytotoxic agents.

Many chemotherapeutic compounds currently in use for treatment of malignant disease are well known for their acute toxicity to the haemopoietic system. Proliferating progenitor cells in the bone marrow are especially vulnerable and their depletion leads to rapid loss of blood cell elements with the serious consequences of infection, haemorrhage and anaemia. These problems can now be largely ameliorated by an intervening transplant of bone marrow or peripheral haemopoietic cells, which allows the opportunity to deliver higher but tolerable doses of chemo- and or radiotherapy. Such a strategy is being increasingly used against a number of refractory malignancies, including leukaemias, lymphomas, multiple myeloma, neuroblastoma and breast cancer (Armitage, 1989; Kessinger & Armitage, 1991; Phillips *et al.*, 1991; Bortin *et al.*, 1992*a*; Marks *et al.*, 1992; Hohaus *et al.*, 1993). Transplant of normal haemopoietic stem cells also offers a means to correct the various genetic diseases of the lymphohaemopoietic system such as sickle cell anaemia, thalassaemia and severe combined immunodeficiency (Rappeport *et al.*, 1983; Barrett & McCarthy, 1990; Brochstein, 1992). Central to the outcome of bone marrow transplantation (BMT) in all these diseases are the relationships between the sensitivity of different host haemopoietic cell subsets towards certain cytotoxic treatments and the potential for repopulation of composite cell subsets derived from the transplanted donor marrow.

Experimental studies in mice have often focused on the importance of transplanting spleen colony-forming units (CFU-S) for rapid repopulation and rescue of recipients from the otherwise lethal acute effects of cytotoxic treatment. However, recent investigations using donor marrow stem cell separation techniques and radiation chimera models have shown that most, if not all, CFU-S cells are limited to the provision of transient short-term haemopoietic support, whereas a more primitive and distinct stem cell subset appears to be responsible for long-term repopulation (Ploemacher & Brons, 1989; Jones *et al.*, 1990; Ploemacher *et al.*, 1993; Down & Ploemacher, 1993). In this respect, quantification of cobblestone area-forming cells (CAFCs) growing in long-term bone marrow cultures has been used to resolve different haemopoietic subsets in sorted marrow cell fractions that correlate with the temporal development of erythroid and leucocyte chimerism in irradiated transplant recipients (Down & Ploemacher, 1993; Ploemacher *et al.*, 1993). The heterogeneous nature of bone marrow stem cells as demonstrated with *in vitro* CAFC and *in vivo* blood chimerism assays has also been investigated under the differential effects of host preparation with the drugs 5-fluorouracil (5-FU) and busulphan (Down & Ploemacher, 1993). In this case both 5-FU and busulphan were toxic to CAFCs developing early in culture (days 5–15) and necessitated early engraftment on behalf of transiently repopulating haemopoietic cells from the transplant. Nevertheless, only busulphan chemotherapy was capable of depleting late- (day 25–35) developing CAFC subsets, and this seemed to be essential for achieving lasting and high levels of donor haemopoietic engraftment. The object of the present study was to extend this experimental approach to investigate other types of alkylating agents, most of which are commonly included in high-dose chemotherapy regimens prior to autologous or allogeneic BMT. These agents were found to have diverse toxicities for the different cell subsets of the haemopoietic hierarchy and offered a useful basis for further comparison of donor blood chimerism *in vivo* with pretransplant marrow CAFC survival as determined *in vitro*.

Correspondence: J.D. Down.
Received 23 March 1994; and in revised form 24 May 1994.

### Materials and methods

### *Animals*

Male and female C57BL 6JIco (B6-*Gpi-1*$^b$ *Gpi-1*$^b$) mice, 12–16 weeks old (IffaCredo, L'Arbresle, France), were used as recipients. Sex-matched C57BL 6J-*Gpi-1*$^a$ *Gpi-1*$^a$ (B6-*Gpi-1*$^a$)

congenic mice were used as a source of donor bone marrow. Animals were housed in approved facilities free of known pathogenic organisms (Sendai, MHV, PVM, GD VII, REO III, EMC, LMC, MVM, K and *Myecobacteria*). Experiments were performed in accordance with the Netherlands Experiments on Animals Act (1977) and the European Convention for the protection of vertebrate animals used for experimental purposes (Strassbourg, 18 March 1986).

### *Treatment*

All drugs were administered intraperitoneally immediately after dissolution in volumes of 0.1 ml per 10 g body weight. Cyclophosphamide (CY) (200 mg $kg^{-1}$; Aldrich-Chemie, Steinheim, Germany), bischloroethylnitrosourea (BCNU) (40 mg $kg^{-1}$; Bristol Laboratories, Syracuse, NY, USA) and isopropylmethane sulphonate (IMS) (100 mg $kg^{-1}$; Paterson Institute of Cancer Research, Manchester, UK) were dissolved in sterile phosphate-buffered saline (PBS). Melphalan (10 and 20 mg $kg^{-1}$; The Wellcome Foundation, London, UK) was dissolved in 16.7% acid alcohol in diluent. Busulphan (50 mg $kg^{-1}$; Sigma, St Louis, MO, USA) and two isomers of dimethylbusulphan (meso-DMB and ±-DMB, 12 mg $kg^{-1}$; Paterson Institute of Cancer Research) were dissolved in 50% dimethylsulphoxide (DMSO) in PBS. The untreated control mice were given either PBS or 50% DMSO/PBS alone. Each treatment group consisted of 8–9 mice, with 3–4 allocated for the CAFC assay and five for bone marrow transplant.

In a separate experiment on melphalan and IMS, the recipient mice were maintained on the non-absorbable antibiotic neomycin sulphate (3.5 g $l^{-1}$; E-Z-EM Rooster, Dordrecht, The Netherlands), administered in drinking water 7 days before and 14 days after treatment. It is estimated that this dose and course of antibiotic treatment completely suppressed the intestinal microflora at the time of treatment (Van der Waaij & Berghuis-de Vries, 1974; Goris *et al.*, 1986*a*) and is capable of protecting mice from radiation-induced gut lethality (Pearson & Phelps, 1981).

### *Determination of haemopoietic subset frequencies* in vitro *(CAFC assay)*

At 24 h after drug treatment femoral bone marrow cells were harvested, pooled from groups of 3–4 mice and plated at limiting dilutions on pre-established and irradiated (20 Gy) bone marrow stromal cultures as previously described (Ploemacher *et al.*, 1989). These cultures allowed growth of haemopoietic precursors under the stromal layer, giving the appearance of cobblestones on phase-contrast microscopy. The frequency of cobblestone area-forming cells (CAFCs) was then determined at various times between 5 and 40 days after overlay. This *in vitro* system provided an estimate of the surviving fraction of CAFCs on different days, which correspond to granulocyte–macrophage colony-forming units (CFU-GM) (CAFC day 5), CFU-S day 12 (CAFC day 10) and the primitive stem cells with long-term repopulating ability (CAFC day 30–40) (Ploemacher *et al.*, 1989, 1991, 1993).

### *Bone marrow transplant and determination of engraftment* in vivo *(Gpi-1 chimerism assay).*

At 24 h after the drug treatments, allocated mice were transplanted i.v. with $10^7$ nucleated bone marrow cells freshly harvested from the tibia and femur of B6-*Gpi-1*$^a$ donor mice. The level of erythroid chimerism in blood samples obtained at different intervals and of chimerism in bone marrow, spleen and thymus at 36 weeks after transplant was determined from glucose phosphate isomerase (*Gpi-1*) electrophoresis as previously described (Van Os *et al.*, 1992).

**Figure 1** Effects of pretreatment with melphalan (●), CY (▲), BCNU (∇) or IMS (■) on (**a**) percentage survival of bone marrow CAFCs on different days and (**b**) development of donor (B6-*Gpi-1*$^a$) blood chimerism after BMT (mean ± 1 s.e.m.). Shown for comparison are data for the effects of 5-FU (□) from Down and Ploemacher (1993).

### Results

### *Toxicity*

Drug doses were first administered on the basis of the maximal tolerance levels in bone marrow reconstituted mice as given in the literature (Floersheim & Ruszkiewicz, 1969; Marsh, 1976). No adverse toxicities were seen in transplanted mice pretreated with 200 mg $kg^{-1}$ CY, 100 mg $kg^{-1}$ IMS and 12 mg $kg^{-1}$ meso- or ±-DMB. BCNU (40 mg $kg^{-1}$)-treated animals also appeared to be in good health throughout the 20 week assay period, but severe renal toxicity had developed by 9 months as evidenced from excessive urine output, elevated blood urea nitrogen (240% of control) and reduced kidney weights (50% of control). Histological examination of kidney tissue showed necrosis and loss of cortical tubular epithelium. Within hours following 50 mg $kg^{-1}$ busulphan, the mice became inactive, hypothermic and prone to convulsions, but by 1 day after treatment they appeared fully recovered. A dose of 20 mg $kg^{-1}$ melphalan allowed survival of mice to 1 day for bone marrow CAFC estimates, but this dose proved to be lethal owing to gut damage at 4 days. The melphalan dose was subsequently lowered to 10 mg $kg^{-1}$, which enabled eight of nine tranplant recipients (89%) to survive for estimation of donor bone marrow engraftment. Melphalan-treated animals also experienced loss of incisors over the period of 2–3 months after treatment, and so powdered food was supplied to avoid problems of malnutrition. A similar problem has been noted previously in mice following treatment with CY and radiation (Pearson & Phelps, 1981; Down & Mauch, 1991).

### *Treatment with melphalan, CY, BCNU, IMS or 5-FU*

The femoral content of CAFCs 5–40 days after overlay in mice treated 24 h earlier with melphalan, CY, BCNU and IMS is shown in Figure 1a as a percentage of normal control values. Also shown are previously published CAFC data on 5-FU (Down & Ploemacher, 1993). Melphalan and 5-FU appeared to have similar effects and were the most toxic to early-developing CAFC subsets (at 5–10 days), with less than 10% survival. Nevertheless, these agents did not affect later developing CAFCs, indicating relative sparing of haemopoietic cells capable of maintained growth *in vitro*. Lower toxicity (>10% survival) was found for early-developing CAFCs following treatment with CY, BCNU or IMS.

Owing to early CAFC depletion in the drug recipients, melphalan and 5-FU had similar effects in promoting an early wave of donor chimerism during the first 10 weeks after transplant (Figure 1b). Recovery of host haemopoiesis after 10 weeks was lower in patients treated with melphalan, as also reflected in the higher toxicity for primitive, late CAFCs (Figure 1a). CY, BCNU and IMS induced low, moderate and relatively high engraftment levels respectively up to 20 weeks post BMT (Figure 1b). In this case BCNU and especially IMS induced engraftment levels that are at variance with the CAFC frequencies shown in Figure 1a.

*Treatment with oral neomycin and melphalan or IMS*

Figure 2 compares two separate experiments on the effects of melphalan and IMS on mice maintained on oral neomycin in order to alleviate the gastrointestinal toxic effects of melphalan. As shown in Figure 2a, CAFC survival after melphalan was much higher in antibiotic-treated recipients, and this is consistent with the corresponding low levels of blood cell chimersim after BMT, as shown in Figure 2b. The effects of IMS on CAFC frequency (Figure 2a) or on donor marrow engraftment (Figure 2b) did not appear to be influenced by neomycin treatment.

The protective effect of intestinal decontamination by neomycin was additionally demonstrated in an experiment in which bone marrow CFU-S appearing on the spleen were measured after 7 days in irradiated (9.5 Gy) secondary recipients. In this case, melphalan without neomycin was able to deplete more than two decades of CFU-S, whereas only 50% of CFU-S were killed in animals maintained on the antibiotic (data not shown).

*Treatment with busulphan or the isomers of DMB*

Figure 3a displays the percentage CAFC survival following treatment with the dimethane sulphonate compounds busulphan and the DMB isomers. These agents proved to be especially toxic to the primitive CAFC subsets that appeared after 28 days, causing cell kill of more than two decades. Both DMB derivatives appeared to be more toxic to CAFCs developing before 28 days than the parent compound. In contrast to the other alkylating agents studied, we also found that the cobblestone cells remaining after busulphan, ±-DMB or meso-DMB were qualitatively abnormal (diminished in number and size), as noted in a previous study on busulphan (Down & Ploemacher, 1993).

The dimethane sulphonate compounds were found to be effective BMT preparative agents for both early and late donor engraftment (Figure 3b). In this case busulphan and ±DMB were similar, while the meso-DMB isomer was less effective.

*Long-term donor type engraftment in blood, bone marrow, spleen and thymus*

The different levels of late blood chimerism seen at 36 weeks in the groups pretreated with melphalan, CY, BCNU, IMS, busulphan and DMB were also reflected in engraftment among other haemopoietic and lymphopoietic cells of the bone marrow, spleen and thymus (Table I). No significant changes in marrow, spleen and thymus cellularity or spleen and thymus weight were observed in these animals at 36 weeks post BMT as compared with untreated controls (data not shown). Peripheral erythrocyte and leucocyte counts were also normal in treated mice.

**Figure 2** Comparison of experiments on mice maintained with or without oral neomycin. Shown are effects of melphalan without (●) and with (○) neomycin (neo) and of IMS without (■) and with (□) neomycin on (**a**) percentage survival of bone marrow CAFC day-types and (**b**) development of donor (B6-*Gpi-1*[a]) blood chimerism after BMT.

**Figure 3** Effects of pretreatment with busulphan (○), meso-DMB (▲) or + -DMB (▼) on (**a**) percentage survival of bone marrow CAFCs on different days and (**b**) development of donor (B6-*Gpi-1*[a]) blood chimerism after BMT.

Table I Chimerism[a] at 36 weeks after different alkylating drugs and BMT in erythrocytes, bone marrow, spleen and thymus

| Drug | Blood | Bone marrow | Spleen | Thymus |
|---|---|---|---|---|
| Melphalan (8)[b] | 11.4 ± 2.2 | 12.9 ± 2.7 | 12.5 ± 2.8 | 15.9 ± 4.3 |
| Cyclophosphamide (4) | 4.0 ± 2.6 | 2.3 ± 2.3 | 2.3 ± 2.3 | 1.3 ± 1.3 |
| BCNU (5) | 26.2 ± 4.0 | 22.4 ± 3.2 | 32.4 ± 2.6 | 16.7 ± 3.1 |
| IMS (5) | 63.0 ± 2.3 | 49.1 ± 2.3 | 48.0 ± 6.7 | 52.3 ± 2.7 |
| meso-DMB (5) | 64.6 ± 8.8 | 52.3 ± 11.3 | 55.0 ± 12.3 | 52.3 ± 11.4 |
| ±-DMB (5) | 92.1 ± 2.3 | 86.4 ± 4.2 | 78.5 ± 2.1 | 92.2 ± 3.6 |
| Busulphan (5) | 78.9 ± 12.8 | 73.8 ± 12.4 | 80.2 ± 8.5 | 67.7 ± 14.2 |

[a]The relative amount of donor-type cells (chimerism) as measured by Gpi-1 phenotyping is given as the mean ± 1 s.e.m. [b]Number of recipients.

### Discussion

The association between bone marrow stem cell heterogeneity and chemosensitivity is a subject of continuing interest in experimental and clinical haematology. It is often held that the susceptibility of the bone marrow to most chemotherapeutic drugs is related to the high proliferative activity of certain haemopoietic cell populations. This is well recognised for the cell cycle-specific effects of the antimetabolite 5-FU against the committed and rapidly proliferating progenitor cell populations that give rise to CFU-GM and early-developing CFU-S (Hodgson & Bradley, 1979; Hodgson et al., 1982). In a previous study (Down & Ploemacher, 1993) we found that this effect corresponds to the marked depletion of the early-developing and cycling CAFC subsets and the early wave of donor erythroid chimerism. The resistance of quiescent primitive stem cells relates in turn to complete survival of late-developing CAFCs and subsequent return of host-type haemopoiesis after BMT. In the present study on alkylating agents, melphalan exhibited similar effects to 5-FU on both early CAFC content and early engraftment to denote a differential cell killing effect on proliferating cells. The acute toxicity that we observed in other tissues of rapid cell renewal, namely the intestinal epithelium and rodent incisors, supports a general susceptibility of cycling cells towards melphalan.

The data obtained from the protective effects of gut decontamination by neomycin on CAFC and CFU-S kill by melphalan provide interesting supplementary information that may also be related to the cycling status of haemopoietic cells. In this case, removal of intestinal aerobic Gram-negative bacteria may indirectly inhibit haemopoietic cell proliferation through reduction of endotoxin levels and thus confer increased resistance to melphalan. This finding is consistent with previous studies showing decreased kill by the S-phase-specific drug hydroxyurea of bone marrow CFU-S and CFU-GM in mice by administration of the antibiotic polymyxin (Goris et al., 1985, 1986b). Since the microbial composition of the intestinal microflora in mice is likely to vary between different laboratories, associated variations in bone marrow stem cell kinetics may hamper direct comparisons of cytostatic drug effects between groups of investigators.

The relatively small effects of CY and BCNU on CAFCs at all times after overlay is at variance with published levels of CFU-S depletion, more than one decade cell kill having been commonly observed with these drug doses (Marsh, 1976). Our finding of greater than 10% CAFC survival following CY treatment was, however, a consistent finding in three other experiments regardless of whether the bone marrow was assayed 6 or 24 h after CY treatment (data not shown). In the present study, the relatively high CAFC survival levels are reflected by the low incidence of early and late donor-type chimerism in CY-pretreated recipients.

In apparent contrast to the findings of our study, Massa et al. (1987) found no donor-type cells in mice pretreated with IMS. However, their study used a lower drug dose of 50 mg $kg^{-1}$ and the 5 day delay to BMT may have rendered IMS less effective in inducing stem cell engraftment. IMS and, to a lesser extent, BCNU induced higher chimerism levels than could be predicted from the magnitude of host CAFC depletion. Such anomalies leave open the interesting possibility that, apart from the eradication of stem cells in the bone marrow at 24 h after treatment, other as yet unknown factors can influence the engraftment of transplanted cells. An IMS-induced stromal defect seems unlikely since this would similarly affect outgrowth of both host and grafted stem cells. Other factors could include a delayed stem cell depletion effect after 24 h or a change in seeding efficiency of the donor cells.

Busulphan is the drug most frequently chosen for treatment of chronic myelogenous leukaemia and has also been increasingly used in recent years as an alternative to total body irradiation in preparative regimens for BMT (Bortin et al., 1992b; Copelan & Deeg, 1992). Of particular interest to the present study is the characteristic toxicity of DMB and the parent busulphan compound against the primitive and quiescent stem cell population, as clearly shown by severe depletion of the late CAFC subset. This concords with provision for long-term donor bone marrow engraftment as previously documented in mice (Massa et al., 1987; Mauch et al., 1988; Lapidot et al., 1989; Leong et al., 1992), rats (Santos & Tutschka, 1974) and humans (Fishleder et al., 1992). Indeed, the low survival of late CAFCs and high levels of long-term engraftment following treatment with busulphan and ±DMB in the present study are similar to total body irradiation given at a dose of between 6 and 7 Gy (Down & Ploemacher, 1993). The diminished quality as well as the quantity of bone marrow CAFCs that we observed after treatment with busulphan and the DMB isomers may certainly confer an additional growth advantage on donor cells as compared with host stem cells *in vivo*. Such an effect can presumably dissociate CAFC frequency from chimerism and may explain the subtle differences in engraftment levels between the two DMB isomers. Further experiments are under way to explore more directly the issue of stem cell quality (as determined by clonal expansion in long-term stroma-supported bone marrow cultures) versus quantity (absolute frequencies as measured in the CAFC assay) in relation to engraftment of donor bone marrow.

While busulphan has been in routine clinical use since the 1950s, relatively little is known of the actual target molecules with which it reacts to inactivate a given cell. The chemical structure of dimethane sulphonates allows for more restricted spacing of the two reactive groups as compared with the other bifunctional alkylating chemotherapeutic agents used in this study, i.e. melphalan, CY and BCNU. Investigations on different analogues of busulphan have shown a remarkable range of biological activities among various cell renewal tissue systems (Berenbaum et al., 1967; Fox & Fox, 1967; Dunn & Elson, 1970; Fox, 1975). These diverse cytotoxic effects have been shown to be related to the distance and orientation of the two alkylating groups, which in turn provides information on the structure and function of critical target (receptor) molecules (Bedford & Fox, 1983; Hartley & Fox, 1986; Fox et al., 1991; Hadfield et al., 1992). Such considerations may be applied in the search for new compounds that have the ability to selectively deplete late-developing CAFCs

(representing resting primitive stem cells) for use in improved BMT conditioning therapy. The recent development of an equivalent CAFC assay system for human marrow (Breems *et al.*, 1994) may prove valuable in bringing this type of treatment closer to a clinical realisation.

We wish to thank Dr John Hadfield for the chemical analysis of the dimethane sulphonate ester compounds used in this study. The technical assistance of Mr Abderaheem Toulab and Miss Wendie de Kreiger is greatly appreciated. This study was partly supported by a grant from the Dutch Cancer Society (GUKC 92-16).

**References**

ARMITAGE, J.O. (1989). Bone marrow transplantation in treatment of patients with lymphoma. *Blood*, **73**, 1749–1758.

BARRETT, J. & MCCARTHY, D. (1990). Bone marrow transplantation for genetic disorders. *Blood Rev.*, **4**, 116–131.

BEDFORD, P. & FOX, B.W. (1983). DNA–DNA interstrand crosslinking by dimethane sulphonate-induced esters. Correlation with linking by dimethane sulphonate acid esters. Correlation with cytotoxicity and antitumour activity in the Yoshida lymphosarcoma model and relationship to chain length. *Biochem. Pharmacol.*, **32**, 2297–2301.

BERENBAUM, M.C., TIMMIS, G.M. & BROWN, I.N. (1967). The relationship between the physico-chemical properties and immunosuppressive effects of an homologous series of sulphonic acid esters. *Immunology*, **13**, 517–522.

BORTIN, M.M., HOROWITZ, M.M. & RIMM, A.A. (1992*a*). Increasing utilization of bone marrow transplantation. Results of the 1988–1990 survey. *Ann. Intern. Med.*, **116**, 505–512.

BORTIN, M.M., HOROWITZ, M.M., GALE, R.P., BARRETT, A.J., CHAMPLIN, R.E., DICKE, K.A., GLUCKMAN, E., KOLB, H.J., MARMONT, A.M., MRSIC, M., SOBOCINSKI, K.A., WEINER, R.S. & RIMM, A.A. (1992*b*). Changing trends in allogeneic bone marrow transplantation for leukemia in the 1980s. *JAMA*, **268**, 607–612.

BREEMS, D.A., BLOKLAND, E.A.W., NEBEN, S. & PLOEMACHER, R.E. (1994). Frequency analysis of human primitive haematopoietic stem cells using a cobblestone area forming cell assay. *Leukemia*, **8**, 1095–1104.

BROCHSTEIN, J.A. (1992). Bone marrow transplantation for genetic disorders. *Oncology*, **6**, 51–58.

COPELAN, E.A. & DEEG, H.J. (1992). Conditioning for allogeneic marrow transplantation in patients lymphohematopoietic malignancies without the use of total body irradiation. *Blood*, **80**, 1648–1658.

DOWN, J.D. & MAUCH, P.M. (1991). The effect of combining cyclophosphamide with total-body irradiation on donor bone marrow engraftment. *Transplantation*, **51**, 1309–1310.

DOWN, J.D. & PLOEMACHER, R.E. (1993). Transient and permanent engraftment potential of murine hematopoietic stem cell subsets: differential effects of host conditioning with gamma radiation and cytotoxic drugs. *Exp. Hematol.*, **21**, 913–921.

DUNN, C.D.R. & ELSON, L.A. (1970). The effect of a homologous series of dimethane-sulphonoxy-alkanes on hemopoietic colony forming units in the rat. *Chem. Biol. Interact.*, **2**, 273–281.

FISHLEDER, A.J., BOLWELL, B. & LICHTIN, A.E. (1992). Incidence of mixed chimerism using busulfan cyclophosphamide containing regimens in allogeneic bone marrow transplantation. *Bone Marrow Transplant.*, **9**, 293–297.

FLOERSHEIM, G.L. & RUSZKIEWICZ, M. (1969). Bone-marrow transplantation after antilymphocyte serum and lethal chemotherapy. *Nature*, **222**, 854–857.

FOX, B.W. (1975). Mechanism of action of methanesulphonates. In *Antineoplastic and Immunosuppressive Agents*, Part II, Sartorelli, A.C. & Johns, D.G. (eds) p. 35. Springer: Berlin.

FOX, B.W. & FOX, M. (1967). Biochemical aspects of the action of drugs on spermatogenesis. *Pharmacol. Rev.*, **19**, 21–57.

FOX, B.W., HADFIELD, J.A. & O'CONNOR, P.M. (1991). Dimethanesulphonate esters in receptor mapping studies. 1. Benzene 1,2-, 1,3- and 1,4-diol, dimethanol and diethanol dimethanesulphonates and anti-tumour activity. *Anti-Cancer Drug Design*, **6**, 71–82.

GORIS, H., DE BOER, F. & VAN DER WAAIJ, D. (1985). Myelopoiesis in experimentally contaminated specific-pathogen-free and germfree mice during oral administration of polymyxin. *Infect. Immunity*, **50**, 437–441.

GORIS, H., DE BOER, F. & VAN DER WAAIJ, D. (1986*a*). Oral administration of antibiotics and intestinal flora associated endotoxin in mice. *Scand. J. Infect. Dis.*, **18**, 55–63.

GORIS, H., DAENEN, S., HALIE, M.R. & VAN DER WAAIJ, D. (1986*b*). Effect of intestinal flora modulation by oral polymyxin treatment on hemopoietic stem cell kinetics in mice. *Acta Haematol.*, **76**, 44–49.

HADFIELD, J.A., FOX, B.W. & CAFFREY, R. (1992). Dimethanesulphonate esters in receptor mapping studies. 2. Antitumour activities of alkyl and alkoxy dimethanesulphonates substituted on a benzene nucleus. *Anti-Cancer Drug Design*, **7**, 263–275.

HARTLEY, J.A. & FOX, B.W. (1986). Cross-linking between histones and DNA following treatment with a series of dimethanesulphonate esters. *Cancer Chemother. Pharmacol.*, **17**, 56–62.

HODGSON, G.S. & BRADLEY, T.R. (1979). Properties of hematopoietic stem cells surviving 5-fluorouracil treatment: evidence of a pre-CFU-S cell. *Nature*, **281**, 381–382.

HODGSON, G.S., BRADLEY, T.R. & RADLEY, J.M. (1982). The organization of hemopoietic tissues as inferred from the effects of 5-fluorouracil. *Exp. Hematol.*, **10**, 26–35.

HOHAUS, S., GOLDSCHMIDT, H., EHRHARDT, R. & HAAS, R. (1993). Successful autografting following myeloablative conditioning therapy with blood stem cells mobilized by chemotherapy plus rhG-CSF. *Exp. Hematol.*, **21**, 508–514.

JONES, R.J., WAGNER, J.E., CELANO, P., ZICHA, M.S. & SHARKIS, S.J. (1990). Separation of pluripotent haematopoietic stem cells from spleen colony-forming cells. *Nature*, **347**, 188–189.

KESSINGER, A. & ARMITAGE, J.O. (1991). The evolving role of autologous peripheral stem cell transplantation following high-dose therapy for malignancies. *Blood*, **77**, 211–213.

LAPIDOT, T., TERENZI, A., SINGER, T.S., SALOMON, O. & REISNER, Y. (1989). Enhancement by dimethyl myleran of donor type chimerism in murine recipients of bone marrow allografts. *Blood*, **73**, 2025–2032.

LEONG, L.Y.W., QIN, S., COBBOLD, S.P. & WALDMANN, H. (1992). Classical transplantation tolerance in the adult: the interaction between myeloablation and immunosuppression. *Eur. J. Immunol.*, **22**, 2825–2830.

MARKS, L.B., HALPERIN, E.C., PROSNITZ, L.R., ROSS, M., VREDENBURGH, J.V., ROSNER, G.L. & PETERS, W. (1992). Post-mastectomy radiotherapy following adjuvent chemotherapy and autologous bone marrow transplantation for breast cancer patients with $\geqslant 10$ positive axillary lymph nodes. *Int. J. Radiat. Oncol. Biol. Phys.*, **23**, 1021–1026.

MARSH, J.C. (1976). The effects of cancer chemotherapeutic agents on normal hematopoietic precursor cells: a review. *Cancer Res.*, **36**, 1853–1882.

MASSA, G., WYLLIE, J.P., PRATT, A.M., MOLINEUX, G. & SCHOFIELD, R. (1987). Marrow repopulation in mice treated with busulphan or isopropyl methane sulphonate and bone marrow. *Br. J. Haematol.*, **66**, 11–14.

MAUCH, P., DOWN, J.D., WARHOL, M. & HELLMAN, S. (1988). Recipient preparation for bone marrow transplantation. I. Efficacy of total body irradiation and busulfan. *Transplantation*, **46**, 205–210.

PEARSON, A.E. & PHELPS, T.A. (1981). Radiation effects on mouse incisor teeth following whole-body doses of up to 16 Gy. *Int. J. Radiat. Biol.*, **39**, 409–417.

PHILLIPS, G.L., SHEPHERD, J.D., BARNETT, M.J., LANSDORP, P.M., KLINGEMANN, H.G., SPINELLI, J.J., NEVILL, T.J., CHAN, K.W. & REECE, D.E. (1991). Busulfan, cyclophosphamide, and melphalan conditioning for autologous bone marrow transplantation in hematological malignancy. *J. Clin. Oncol.*, **9**, 1880–1888.

PLOEMACHER, R.E. & BRONS, N.H.C. (1989). Separation of CFU-S from primitive cells responsible for reconstruction of the bone marrow hemopoietic stem cell compartment following irradiation: evidence for a pre-CFU-S cell. *Exp. Hematol.*, **17**, 263–266.

PLOEMACHER, R.E. & VAN DER SLUIJS, J.P., VOERMAN, J.S.A. & BRONS, N.H.C. (1989). An *in vitro* limiting dilution assay of long-term repopulating hemopoietic stem cells in the mouse. *Blood*, **74**, 2755–2763.

PLOEMACHER, R.E., VAN DER SLUIJS, J.P., VAN BEURDEN, C.A.J., BAERT, M.R.M. & CHAN, P.L. (1991). Use of limiting-dilution type long-term marrow cultures in frequency analysis of marrow-repopulating and spleen colony-forming hemopoietic stem cells in the mouse. *Blood*, **78**, 2527–2533.

PLOEMACHER, R.E., VAN DER LOO, J.C.M., VAN BEURDEN, C.A.J. & BAERT, M.R.M. (1993). Wheat germ agglutinin affinity of murine hemopoietic stem cell subpopulations is an inverse function of their long term repopulating ability *in vitro* and *in vivo*. *Leukemia*, **7**, 120-130.

RAPPEPORT, J.M., SMITH, B.R., PARKMAN, R. & ROSEN, F.S. (1983). Application of bone marrow transplantation in genetic diseases. *Clinics in Haematol.*, **12**, 755-773.

SANTOS, G.W. & TUTSCHKA, P.J. (1974). Marrow transplantation in the busulfan-treated rat: preclinical model of aplastic anemia. *J. Natl Cancer Inst.*, **53**, 1781-1785.

VAN DER WAAIJ, D. & BERGHUIS-DE VRIES, J. (1974). Oral dose and faecal concentration of antibiotics during decontamination in mice and in a patient. *J. Hyg. Camb.*, **73**, 197-203.

VAN OS, R., KONINGS, A.W.T. & DOWN, J.D. (1992). Radiation dose as a factor in host preparation for bone marrow transplantation across different genetic barriers. *Int. J. Radiat. Biol.*, **61**, 501-510.